# Role of Adiponectin and Its Receptors in Cancer

**DOI:** 10.7497/j.issn.2095-3941.2012.04.001

**Published:** 2012-12

**Authors:** Stephanie Obeid, Lionel Hebbard

**Affiliations:** Storr Liver Unit, Westmead Millennium Institute, PO Box 412, Darcy Road, Westmead, NSW 2145, Australia

**Keywords:** adiponectin, cancer, signaling

## Abstract

Adiponectin (APN), a novel hormone/cytokine derived from adipocyte tissue, is involved in various physiological functions. Genetics, nutrition, and adiposity are factors contributing to circulating plasma concentrations of APN. Clinical correlation studies have shown that lower levels of serum APN are associated with increased malignancy of various cancers, such as breast and colon cancers, suggesting that APN has a role in tumorigenesis. APN affects insulin resistance, thus further influencing cancer development. Tumor cells may express receptors for APN. Cellular signaling is the mechanism by which APN exerts its host-protective responses. These factors suggest that serum APN levels and downstream signaling targets of APN may serve as potential diagnostic markers for malignancies. Further research is necessary to clarify the exact role of APN in cancer diagnosis and therapy.

## Introduction

Adipose tissue is responsible for the release of proteins involved in the homeostasis of glucose, lipids, and systemic inflammation. Adiponectin (APN) is produced solely and most abundantly by adipose tissues^[^^1^^]^ and plays an important role in physiological responses to disease, such as inflammation^[^^2^^]^. Glucose and fatty acid metabolism are also modulated by APN^[^^3^^]^, and decreased plasma concentrations of APN are linked to insulin resistance, type II diabetes^[^^4^^]^, and atherosclerosis^[^^5^^]^. Recent findings have associated APN with cancer progression, which will be reviewed in this article.

## Adiponectin Structure

APN, which comprises 244 amino acids that represent a full 30 kDa-long protein^[^^6^^]^ is encoded on human chromosome 3q27^[^^7^^]^. APN consists of three domains: single peptide, collagen-like motif, and globular domain. Adipokine APN is highly abundant in human serum and is secreted by adipose tissue in an amount inversely proportional to the body mass index (BMI)^[^^8^^]^. Full-length APN exists in three forms in human serum, namely, as low molecular weight (LMW) trimer, as middle molecular weight hexamer that forms through the self-association of two trimers, and as high molecular weight (HMW) multimer^[^^9^^]^. HMW APN is yet to be structurally characterized. Nevertheless, studies have suggested that murine and bovine APN are octadecameric in nature^[^^10^^]^, whereas HMW APN derived from humans comprises multiple species with lengths ranging from 18 mers to 30 mers or longer^[^^9^^]^. A proteolytic cleavage product of APN, which is known as globular APN, has been shown to circulate in human plasma^[^^11^^]^. APN circulates in human plasma in concentrations ranging from 3 to 30 µg/mL and contributes to 0.05% of the total plasma protein^[^^1^^]^.

Post-translational modifications of APN are crucial in the formation of HMW oligomers, as well as in the receptor-binding capabilities and biological actions of APN^[^^12^^,^^13^^]^. Studies have suggested that HMW APN is the most biologically active form of APN, and HMW APN is associated with the improvement of insulin sensitivity after treatment with thiazolidinedione^[^^14^^]^.

## Adiponectin Receptors

Three APN receptors are currently identified. AdipoR1 and AdipoR2 have distinct affinities for the various circulating forms of APN^[^^15^^]^ and consist of seven transmembrane regions, which comprise an internal N-terminal region and an external C-terminal region. AdipoR1 is ubiquitously expressed and is highly present in skeletal muscle, and to a lesser degree, on endothelial cells. AdipoR1 binds globular APN with high affinity and full-length APN with low affinity^[^^16^^]^. AdipoR2 is abundantly expressed in the liver and binds globular and full-length APN with moderate affinity^[^^17^^,^^18^^]^. Studies have shown that AdipoR1 and -R2 can transduce APN-binding interactions through adaptor protein containing pleckstrin homology domain, phosphotyrosine binding domain, and leucine zipper motif (APPL-1). APPL-1 interacts with APN receptors in mammalian cells and is responsible for downstream signaling events, such as lipid oxidation and glucose uptake^[^^19^^]^. The third known receptor capable of binding APN is T-cadherin, a glycosyl-phosphatidylinositol (GPI) receptor that lacks a transmembrane domain which is located on the cellular surfaces of endothelial, epithelial, and smooth muscle cells. T-cadherin is involved in cell–cell interactions and signaling via calcium-dependent mechanisms; T-cadherin also binds to hexameric and HMW forms of APN, but not to trimeric or globular APN^[^^12^^]^. A recent study demonstrates the importance of this interaction and shows that the cardio protective functions promoted by APN are dependent on T-cadherin^[^^20^^]^. GPI-linked T-cadherin may function indirectly with AdipoR1 and -R2 to facilitate APN signaling.

## Adiponectin Signaling Pathways in Cancer

Numerous signaling pathways are utilized by APN in physiological responses. APN exerts its effects via 5′ adenosine monophosphate-activated protein kinase (AMPK), mammalian target of rapamycin (mTOR), phosphatidylinositol 3-kinases/protein kinase B (PI3K/Akt), mitogen activated protein kinase (MAPK), signal transducer and activator of transcription 3 (STAT-3), nuclear factor kappa-light-chain-enhancer of activated B cells (NF-κB) and the sphingolipid metabolic pathway.

The majority of APN signaling in cancer is exerted through AMPK. Increased concentrations of adenosine monophosphate (AMP), calcium-dependent kinases, APPL-1, and Ser/Thr liver kinase B1 (LKB1) contribute to AMPK activation^[^^21^^]^. LKB1 expression in breast cancer cell lines is increased by APN, which results in AMPK activation and inhibition of tumor cell adhesion and migration^[^^22^^]^. AMPK interferes with cellular growth signaling through mTOR, thus inhibiting the promotion of carcinogenesis. AMPK prevents fatty acid synthesis by blocking acetyl-CoAcarboxylase (ACC) and fatty acid synthase (FAS), which is mediated by sterol regulatory element binding protein 1c (SREBP-1c). AMPK also promotes growth arrest and apoptosis via increased p53 and p21 expression, respectively^[^^23^^]^.

Studies have shown that APN affects PI3K/Akt signaling. Growth factors activate PI3K, which results in the phosphorylation of Akt and effector molecules that promote cellular growth and proliferation. Akt is also capable of inhibiting tuberous sclerosis protein 2, which neutralizes the effects of activated AMPK. APN treatment of breast and colorectal cancer cell lines decreases the phosphorylation of PI3K and Akt^[^^24^^]^. In the context of colorectal cancer, the treatment of cell lines with APN resulted in AMPK activation and suppression of mTOR pathway, thus inhibiting cancer cell growth^[^^25^^]^.

The superfamily of MAPKs involves c-Jun N-terminal kinases (JNK), p38 and extracellular signal-regulated kinases (ERK1/2). The effects of p38 and JNK on cellular proliferation are variable, whereas that of ERK1/2 are mitogenic in nature^[^^26^^]^. The application of APN on a hepatocellular carcinoma cell line resulted in increased JNK activation and subsequent apoptosis via caspase-3^[^^27^^]^. *In vitro* studies on endometrial and breast cancer cell lines showed that APN inhibited ERK1/2 signaling, thus resulting in decreased cellular viability^[^^28^^,^^29^^]^. Moreover, the treatment of MCF-7 breast cancer cell lines with APN decreased c-myc, cyclin D, and Bcl-2 levels, and as well increased p52 and Bax expression, thus resulting in cell cycle arrest^[^^29^^]^.

The systemic effects of STAT-3 are modulated by APN in cancer progression. Adipocytokine- and cytokine-mediated phosphorylation of Janus kinase subunits results in the activation of STAT-3, as well as in the subsequent regulation of cellular proliferation and differentiation. The dysregulation of this pathway may result in cancer progression^[^^30^^]^. Studies have shown that the treatment of prostate and liver cancer cell lines with APN results in decreased phosphorylation of STAT-3^[^^31^^,^^32^^]^.

Wnt signaling has been implicated in various cancers. Wnt activates signaling by binding to the cell surface receptor, frizzled, to inactivate glycogen synthase kinase-3β (GSK3β) and to promote the accumulation of β-catenin in the cellular nucleus^[^^33^^]^. APN treatment of MDA-MB-231 breast cancer cells inhibits the phosphorylation of GSK3β, resulting in the degradation of β-catenin and subsequently causes the decrease of cyclin D1 expression. APN exerts its effects in this context via induction of Wnt inhibitory factor-1, a molecule that can downregulate β-catenin expression^[^^34^^]^.

Recent data have shown that APN and T-cadherin interactions can influence tumor blood vessel growth and subsequent tumor aggressiveness^[^^35^^]^. The molecular mechanism by which this interaction affects blood vessel growth has not yet been elucidated. T-cadherin signaling functions that are independent of APN binding have been described in cancer. T-cadherin has been implicated in different types of human cancers, in which gene expression has been silenced by methylation. The downregulation of T-cadherin in human mammary cancer and glioma, together with forced overexpression, suggests that T-cadherin is a tumor suppressor^[^^35^^,^^36^^]^. In addition, T-cadherin expression can either influence or restrict the invasive potential of liver and squamous cell carcinoma cancer cell lines, respectively^[^^37^^,^^38^^]^. Studies implicating APN and T-cadherin interactions in these models are warranted.

## Adiponectin and Cancer

Circulating plasma concentrations of APN are inversely related to increased risks of malignancy. Studies have shown that decreased levels of APN are present in patients with breast^[^^39^^]^, endometrial^[^^40^^]^, prostate^[^^41^^]^, gastric^[^^42^^]^, liver^[^^43^^]^, pancreatic^[^^44^^]^, hematological^[^^45^^,^^46^^]^, and colon cancers^[^^47^^]^. We will consider in detail the association of APN with specific cancer types.

### Breast cancer

Obesity is a risk factor for the development of breast cancer. In particular, studies have shown that obese individuals have decreased serum APN concentrations, thus resulting in an increased risk of post-menopausal breast cancer^[^^48^^]^.

Kang et al.^[^^49^^]^ showed that the addition of APN to the cell line MDA-MB-231 resulted in growth arrest and apoptosis in breast cancer cells. APN can exert growth arrest and apoptosis through the β-catenin-Wnt pathway. The increased nuclear translocation of β-catenin and the overexpression of cyclin D1 are evident in multiple cancers^[^^50^^]^. GSK-3β promotes the proteolysis of β-catenin by phosphorylation at N-terminals^[^^51^^]^. Akt can phosphorylate and inactivate GSK-3β, thereby facilitating cellular stabilization and increased concentrations of β-catenin^[^^33^^]^. Treatment of the MDA-MB-231 breast cancer cell line with APN resulted in the phosphorylation of Akt and in the deactivation of GSK-3β, which subsequently decreased β-catenin expression as well as the transcriptional target of β-catenin, cyclin D1^[^^52^^]^. This mechanism may be cell-specific as the above effects were not observed in the T47D cell line.

Other studies have shown that the addition of APN can inhibit breast cancer cell line responses to growth factors in serum, thus potentially preventing tumor growth^[^^49^^]^. The precise mechanisms underlying APN and its ability to exert anti-cancer effects are yet to be elucidated. Studies have shown that the treatment of MCF-7 breast cancer cell lines with APN results in AMPK activation. AMPK is an intracellular signaling protein, which responds to stress factors caused by energy depletion signals. AMPK is activated when cellular adenosine triphosphate is low and when the AMP level is high, such as in glucose deprivation, hypoxia, and oxidative stress. AMPK exerts its effects by activating catabolic pathways, such as fatty acid oxidation^[^^25^^]^. Increased FAS is characteristic of malignant breast cancer. FAS inhibition through the use of metformin, a drug used for the treatment of diabetes, activates AMPK and suppresses FAS gene expression. FAS inhibition also inactivates ACC, thereby resulting in decreased lipogenesis and synthesis of malonyl-CoA, to promote fatty acid oxidation^[^^53^^]^. Recently, a mouse model of breast cancer revealed that the use of APN receptor peptide agonists can activate AMPK and reduce tumor growth^[^^54^^]^.

Studies have shown that APN levels affect the phenotypic characterization of tumors in breast cancer. Decreased serum APN concentrations have been associated with a higher risk of breast cancer^[^^55^^,^^56^^]^ and aggressive tumors characterized by increased size, histological score, and estrogen receptor negativity^[^^57^^]^. These attributes are seen in estrogen/progesterone receptor negative breast cancers and suggest that decreased APN may be caused by the deregulation of sex steroid homeostasis^[^^58^^]^. The role of APN in preventing breast tumorigenesis is intertwined with endocrine and paracrine factors. In the context of breast cancer, tumor cells and adipocytes are in close proximity. APN inhibits aromatase activity in adipocytes, which is responsible for the conversion of steroids to estrogens^[^^59^^]^. This activity is mediated by LKB1 and AMPK, which results in decreased estrogen release. This reduction in estrogen receptor stimulation may impair tumor survival^[^^60^^]^.

The examination of the role of APN in breast cancer using experimental mouse models suggests that it may play a role in modulating tumor growth. In the mouse mammary tumor virus–polyoma middle T-antigen (MMTV–PyMT) model, APN knockout mice presented with decreased tumor growth rate and angiogenesis, and increased apoptosis and enhanced metastasis, suggesting a role of APN in modulating angiogenesis during tumor formation^[^^61^^]^. Similarly, in T-cadherin knockout mice bred into the MMTV–PyMT experimental model, breast tumor growth was delayed, and tumors showed reduced blood vessel density, and greater tumor hypoxia with aggressive pathology, as evidenced by metastasis to the lungs. APN was tethered to the tumor vasculture by T-cadherin in wild-type mice, which was absent in T-cadherin knockout mice^[^^35^^]^. These data suggest that APN and T-cadherin interactions are important for regulating breast tumor angiogenesis and metastatic potential.

### Colorectal cancer

Obesity is a risk factor for the development of colorectal cancer (CRC). Studies have shown that APN concentrations in serum are diminished in CRC, specifically in male patients^[^^47^^]^. APN receptors AdipoR1 and -R2 are expressed on the surface of both the carcinoma and normal colonic tissue. Furthermore, despite the decline in APN serum concentrations, the surface expression of AdipoR1 and -R2 is greater in cancerous colonic tissue. This compensatory mechanism is induced by decreased APN concentrations^[^^62^^]^.

The serum levels of APN influence the pathological characteristics of colorectal tumors. A decline in the concentration of circulating APN has been shown to increase colonic adenomas in Japanese patients. This study revealed an inverse relationship between APN and the number and size of tumors, suggesting that APN plays a protective role in cancer progression^[^^63^^]^. A recent study has shown that AdipoR2 is positively associated with tumor, node, and metastasis staging in CRC^[^^64^^]^. Thus, APN might be involved in both the initial and progressive stages of CRC.

The use of CRC cell lines has assisted in clarifying the signaling pathways mediated by APN in disease pathogenesis. APN suppresses CRC proliferation in HCT116, HT29, and LoVo cell lines at the G_1_/S phase of the cell cycle, while increasing the expression of cyclin dependent kinases, such as p27 and p21^[^^65^^]^. The same study revealed that APN stimulated the phosphorylation of AMPK and showed that the inhibition of AMPK resulted in a diminished effect of APN on the proliferation of CRC cells. AdipoR1 and -R2 facilitated such activity because the knockdown of these receptors resulted in increased CRC cell proliferation^[^^65^^]^. Other studies have shown that APN-mediated AMPK activation signaling resulted in the inhibition of the mTOR pathway in CRC cell lines to suppress cancer cell growth^[^^25^^]^. These studies suggest that APN may inhibit the proliferation of CRC cells via AMPK and mTOR signaling^[^^65^^]^.

APN concentrations are inversely related to adiposity and insulin resistance, which are related to increased risks of developing CRC. Experimental models have shown that APN knockout mice are more susceptible to developing CRC compared with wild-type mice. APN null mice are characterized by increased severity of symptoms and increased number and area of tumors, as well as extensive inflammation and immune cell infiltration. These APN null mice also exhibited higher phosphorylated STAT-3, as well as cyclooxygenase-2, which are important in driving metastasis and inflammation^[^^66^^]^. Other studies have indicated that APN suppresses CRC development under a high-fat diet. Fujisawa et al.^[^^67^^]^ showed that APN knockout mice on a high-fat diet had an increased number of colonic polyps, as well as higher colonic epithelial cell proliferation rates, in comparison to APN knockout mice on a basal diet. Their work also showed that APN exerts its host-protective effects through the suppression of colonic epithelial cell proliferation via the inhibition of the mTOR pathway. These experiments indicate the broad biological activity of APN in the context of colorectal carcinogenesis.

A loss of heterozygosity in tumor suppressor genes is characteristic of many cancers such as CRC. The location of T-cadherin on chromosome 16q is suggestive of its tumor-suppressive functions. Toyooka et al.^[^^68^^]^ reported that T-cadherin expression in cancers is silenced due to the hypermethylation of the CDH13 gene at the 5′ end. Hibi et al.^[^^69^^]^ also showed that hypermethylation of CDH13 occurs in the early stages of CRC, which was detected in 17 out of 35 primary CRC tumors, suggesting that T-cadherin may act as a colon cancer suppressor gene. No study to date has examined the role of APN and T-cadherin interactions in CRC.

### Liver cancer

Studies have shown that APN is associated with liver carcinogenesis. In a Japanese case-control study, higher serum concentrations of LMW APN was associated with decreased risks of liver cancer following the assessment of lifestyle habits such as smoking and alcohol consumption, as well as BMI^[^^70^^]^. *In vitro* studies have mimicked such findings, considering APN exerts anti-malignancy effects in hepatocellular carcinoma (HCC) cell lines, HepG2 and Huh7, via leptin inhibition^[^^31^^]^.

Obesity and chronic hepatitis C (CHC) are two risk factors for the development of HCC. Studies have shown that high-APN serum concentrations in patients with CHC have an increased risk of HCC, suggesting that APN plays an oncogenic role in fibrotic livers. This may be caused by the downregulation of APN receptors in the liver. However, more experimental evidence is required to clarify this mechanism^[^^43^^]^. In contrast, another study showed that serum APN concentrations are related to the development of liver fibrosis in the context of the hepatitis C virus, but not for the prevalence of HCC in patients with CHC^[^^71^^]^.

APN is implicated in reducing growth of HCC. Man et al.^[^^72^^]^ utilized an orthotoptic liver tumor nude mouse model to show that APN treatment significantly decreased tumor growth, as well as the occurrence of lung metastasis. APN has also been shown to decrease hepatic stellate cell and macrophage infiltration. The effects of APN in this model are due to the inhibition of tumor angiogenesis and the downregulation of the Rho kinase/IFN-inducible protein 10/matrix metalloproteinase-9 signaling pathway.

### Prostate cancer

Decreased serum levels of APN have been implicated in prostate cancer and its progression. A 70% reduction rate in prostate cancer risk was noted in men with high-APN serum concentrations, independent of lifestyle and age factors^[^^73^^]^. The same study revealed that tumor samples derived from the prostate expressed less APN receptors than those of non-tumor tissue, suggesting that the loss of APN receptor expression is associated with increased cancer formation^[^^73^^]^. Goktas et al.^[^^41^^]^ reported that plasma APN levels were significantly lower in patients with prostate cancer than those in controls. Moreover, APN levels were lower in groups with malignant cancer than those in groups with organ-confined prostate cancer. These studies suggest that decreased plasma APN levels are associated not only with increased risks of prostate cancer but also with the extent of aggressiveness.

Prostatic cancer cell lines have been employed to understand the signaling mechanisms regulated by APN and disease progression. Barb et al.^[^^74^^]^ showed that APN signals through AMPK in phosphatase and tensin homolog (PTEN)-deficient LNCaP prostate cancer cells due to a deletion on chromosome 10. This study showed that APN activated mTOR activity, as demonstrated by the phosphorylation of downstream targets p70 S6 kinase and ribosomal protein S6. The APN-mediated stimulation of mTOR occurred via the PI3k/Akt axis, and the PTEN status of the cells defined the pathways that predominate over mTOR activation. These data suggest that elevated levels of APN in the presence of PTEN loss can promote prostate cancer growth.

### Endometrial cancer

Adiposity and a high BMI are risk factors in the development of endometrial cancer, predominantly in post-menopausal women. Decreased APN concentrations are linked to the increased risk of endometrial cancer in females below the age of 65^[^^40^^]^. Moon et al.^[^^75^^]^ showed that AdipoR1 expression is greater than that of AdipoR2 in human endometrial cancer tissue. They reported that APN suppresses cancer cell proliferation *in vitro*, as mediated by AdipoR1 and -R2. This phenomenon occurs via increased expression of LKB1, a molecule required for the APN-mediated activation of the AMPK/S6 pathway and for the subsequent control of cellular proliferation and invasion.

Studies have shown that APN levels are inversely associated with cancer of the endometrium^[^^76^^]^. Cong et al.^[^^28^^]^ utilized the human endometrial carcinoma cell lines HEC-1-A and RL95-2 to show that APN treatment decreases proliferation mediated by cell cycle arrest at the G_1_/G_0_ phase and the activation of apoptosis. Two signaling mechanisms were proposed. In the HEC-1-A cells, APN increased the phosphorylation of AMPK in the short term and activated Akt while decreasing cyclin D1 expression after prolonged exposure. The RL95-2 cell line exhibited a different behavior as no modulation of Akt or cyclin D1 occurred. However, a decrease in p44/p42 MAPK and cyclin E2 was observed. These experiments suggest that APN comprises inhibitory effects in endometrial cancer through multiple signaling pathways.

### Pancreatic cancer

Obesity, type II diabetes, and insulin resistance are considered risk factors for the development of pancreatic cancer, notably in men. Studies have shown that decreased APN levels are associated with increased risks of pancreatic cancer in male smokers^[^^44^^]^. Another study noted increased levels of serum APN following cancer development, which may be a compensatory mechanism for inflammation and weight loss due to cancer cachexia^[^^77^^]^. This increase in serum APN during cancer may be a compensatory increase due to the downregulation of APN receptors corresponding to a reduction in downstream signaling pathway activity^[^^77^^]^.

### Gastric cancer

Plasma APN levels have been found associated with the development of gastric cancer. Ishikawa et al.^[^^42^^]^ showed that APN serum levels were lower in patients with gastric cancer compared with healthy controls. Moreover, APN was negatively correlated with the size and depth of the tumor, as well as tumor stage. Other studies investigated the role of AdipoR1 and -R2 in cancer prognosis. Cancer cells deficient in AdipoR1 metastasized to the lymphatic system and the peritoneal cavity^[^^78^^]^. Atani et al.^[^^79^^]^ analyzed mRNA levels of AdipoR1 and -R2 in human gastric cancers and reported decreased expression of both receptors, which was attributed to transforming growth factor beta (TGF-β) as a mechanism to escape the protective effects of APN.

## Hematological cancer

APN has been linked to leukemia, lymphoma, and myeloma in individuals with above-average BMI. APN is also linked to malignancies of the myeloid lineage, which include acute myeloblastic leukemia^[^^46^^]^, myelodisplastic syndrome (MDS)^[^^80^^]^, and myeloproliferative diseases, such as chronic myelogenous leukemia (CML)^[^^81^^]^. APN is reported to have an inhibitory effect on myeloid cell proliferation. Studies have shown that AdipoR1 expression was higher in two CML cell lines, whereas AdipoR2 was unchanged. These studies suggest that APN receptors may play a yet unknown role in the pathogenesis of these diseases^[^^82^^]^.

Decreased serum APN is related to MDS, which is a condition comprising defects in hematopoiesis that fosters the development of acute myeloid leukemia^[^^83^^]^. Variations in MDS were observed for different APN levels, where individuals with refractive anemia had higher APN levels than those with more severe anemia. Dalamaga et al.^[^^80^^]^ showed that HMW APN presented an inverse relationship with MDS, in which insulin-like growth factor 1 was positively associated with disease manifestation. Low APN levels have been found associated with CML patients. Individuals undergoing interferon treatment had higher APN concentrations in serum than untreated patients, which may be due to a positive regulation of APN by interferon, as well as the inhibition of pro-inflammatory cytokines in the pathogenesis of disease^[^^81^^]^.

An increased risk of myeloma is associated with decreased levels of APN. Data generated from human studies and experimental models suggest that reduced APN facilitates myeloma progression, thus playing a suppressive role in disease pathogenesis^[^^84^^]^. APN-null mice with myeloma exhibited decreased myeloma cell apoptosis. An increase in circulating APN by the apolipoprotein mimetic peptide L-4F reversed this condition, thus preventing the progression of myeloma bone disease^[^^84^^]^. In view of the anti-inflammatory properties of APN, decreased APN levels in myeloma malignancy are hypothesized to be associated with increased IL-6 and tumor necrosis factor alpha (TNF-α)production^[^^85^^]^.

With regards to chronic lymphoid leukemia (CLL), studies showed that a reduction in APN levels is correlated with disease progression^[^^81^^]^. In contrast, other studies showed that no significant difference in HMW APN levels existed in individuals with B-cell CLL^[^^86^^]^. One group reported that APN had an inverse relationship with markers of disease severity, such as CD38-positive CLL cells and total peripheral blood lymphocyte count. Consequently, APN was positively correlated with the vascular endothelial growth factor (VEGF). This study also showed that CLL cells highly expressed both AdipoR1 and -R2, but had low APN gene expression^[^^87^^]^, suggesting that limited APN fosters disease progression.

APN has also been implicated in the progression of non-Hodgkin’s lymphoma (NHL) in adults and children, as well as Hodgkin’s lymphoma. Patients had higher APN levels than healthy controls. The serum APN levels of adult NHL patients are correlated with IL-10, which is an indicator of poor prognosis^[^^88^^]^. APN has been suggested to have different mechanisms of action with respect to NHL cells and may exert its effects directly via AdipoR1 and -R2 because both are present on the surface of NHL tissues, which may then promote oncogenic signaling. Alternatively, APN levels may affect the levels of other circulating cytokines as seen by the upregulation of IL-10 and the downregulation of TNF-α, which are important in NHL progression^[^^45^^]^ (**Table 1** and **Table 2**).

**Table 1:** *In vivo* models supporting the role of APN in cancer.

Cancer	Model	Reference
Breast cancer	Nude mouse model; MDA-MB-231 cells treated with and without APN	Wang et al.^[^^52^^]^
	PyMT oncogene expressed by MMTV bred into the APN background	Denzel et al.^[^^61^^]^
Colorectal cancer	Syngeneic mouse model; CRC cell line MCA38 injected into APN-null mice and wild-type mice on a high-fat diet	Moon et al.^[^^89^^]^
	Spontaneous tumor progression; Azoxymethane injections weekly in APN-null mice and wild-type mice	Nishihara et al.^[^^90^^]^
	Chronic inflammation-induced CRC; three cycles of dextran sulfate sodium administration into APN^-/-^ mice and wild-type mice, followed by weekly doses of 1,2-dimethylhydrazine for 12 weeks, followed by 3 cycles of DSS	Saxena et al.^[^^66^^]^
Liver cancer	Orthotoptic liver cancer model; MHCC97L cells subcutaneously injected into nude mice	Man et al.^[^^72^^]^
	Nude mouse xenograft model of HCC; subcutaneous injection of HepG2 cells, followed by intraperitoneal injections of leptin and adiponectin	Sharma et al.^[^^31^^]^
Hematological cancer	Multiple myeloma model; wild-type and APN-null mice intravenously treated with 5TGM1-GFP cells	Fowler et al.^[^^91^^]^
Gastric cancer	Nude mouse model; subcutaneous injection of AZ521 cells, followed by APN intratumoral injection	Ishikawa et al.^[^^92^^]^

**Table 2:** Clinical studies on the role of APN in human cancer.

Cancer	APN serum concentration
Breast cancer	Decreased ^[^^39^^,^^48^^,^^55^^-^^57^^]^
Colorectal cancer	Decreased ^[^^47^^,^^63^^,^^93^^]^
Pancreatic cancer	Decreased ^[^^44^^,^^73^^,^^94^^]^
Endometrial cancer	Decreased ^[^^40^^,^^76^^,^^95^^]^
Prostate cancer	Decreased ^[^^41^^,^^73^^]^
Gastric cancer	Decreased ^[^^42^^]^
Liver cancer	Decreased ^[^^43^^]^
Hematological cancer	Decreased ^[^^46^^,^^80^^,^^81^^,^^86^^]^

## Conclusion

APN is linked to obesity, the metabolic syndrome, insulin resistance, type II diabetes, inflammation and various cancers. In certain cancers, such as colorectal, breast, and liver cancers, limited APN promotes tumor growth, suggesting that signaling mediated by APN may be amenable to targeted therapy. Nevertheless, the exact role of APN in other neoplasias remains to be clarified. The role of APN may be dependent on the host tissue environment because it can impede or promote the tumorigenic actions of APN. Further studies on the role of APN in cancer may facilitate the development of new therapeutic targets. Nevertheless, the overarching theme is that an improvement in the western sedentary lifestyle is important in preventing obesity-related cancers.
